# CD320 expression is increased in breast cancer stem cells but does not promote their expansion or alter key histone methylation marks

**DOI:** 10.17912/micropub.biology.001696

**Published:** 2025-09-04

**Authors:** Christine Carney, Lisa Jenkins, Connor Jewell, Rachel Carter, Puneet Mann, Binwu Tang, Senthil Muthuswamy, Lalage Wakefield

**Affiliations:** 1 National Cancer Institute, Bethesda, Maryland, United States

## Abstract

Several lines of evidence suggest a potential link between stemness and vitamin B12, which contributes to S-adenosylmethionine (SAM) generation and hence methylation-dependent epigenetic control. Here we found that the vitamin B12 receptor, CD320, is significantly increased in cancer stem cells (CSCs) in models of triple-negative breast cancer *.* We hypothesized that elevated CD320 expression promotes CSC expansive self-renewal via increased SAM-mediated histone H3K4 and H3K36 trimethylation, which were previously implicated in stemness and plasticity. However, we found that modulation of CD320 expression had no effect on SAM, H3K4me3 and H3K36me3 levels, or on CSC expansion *in vitro* .

**Figure 1. CD320 expression is increased in breast cancer stem cells but does not promote their expansion or alter key histone methylation marks:**
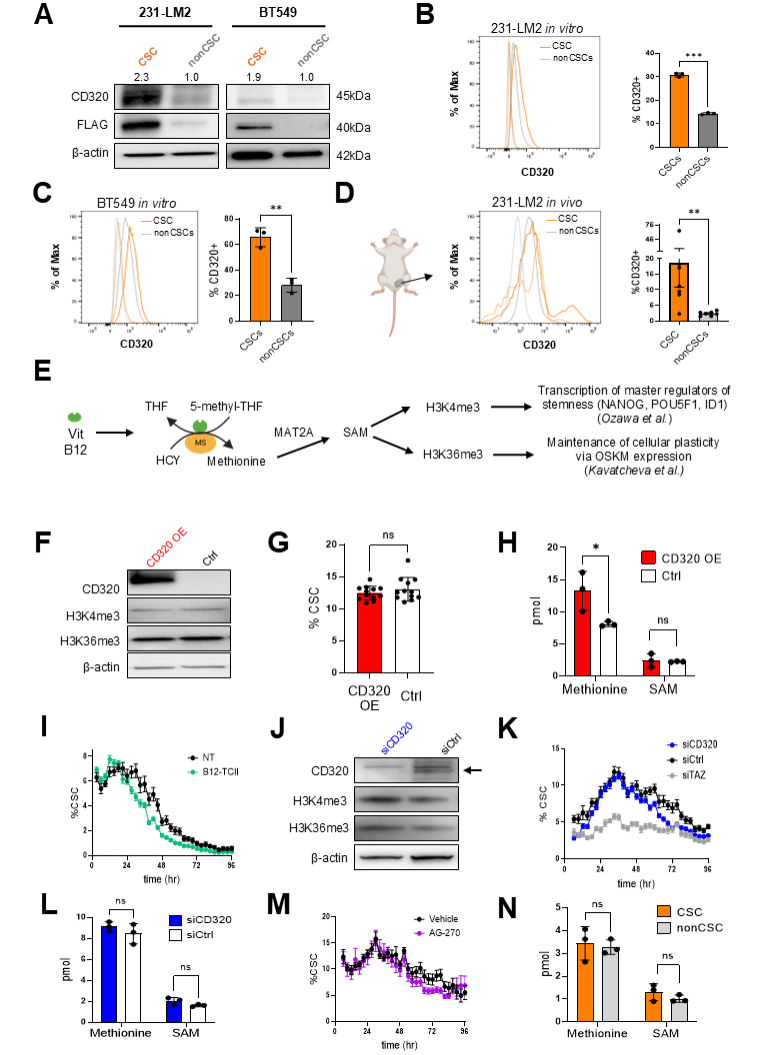
A. Western blot analysis of total CD320 protein expression in sorted 231-LM2 and BT549 CSCs and nonCSCs. FLAG detects the FLAG-tagged dsmCherry of the CSC reporter. Values shown above the blot reflect the CD320 signal normalized to β-actin, normalized to nonCSC values. B. Flow cytometric analysis of surface CD320 expression in unsorted cultures of 231-LM2 CSCs and nonCSCs. Dotted lines show isotype control staining for the respective populations. Unpaired t test, ***p < 0.0005. C. Flow cytometric analysis of surface CD320 expression in unsorted cultures of BT549 CSCs and nonCSCs. Dotted lines show isotype control staining for the respective populations. Paired t test, **p = 0.001. D. Flow cytometric analysis of surface CD320 expression in CSCs and nonCSCs dissociated and sorted from 231-LM2 mammary fat pad tumors (n = 7 tumors). Dotted lines show isotype control staining for the respective populations. Mann-Whitney test, **p > 0.01. E. Schematic showing the intracellular function of vitamin B12, which is a necessary co-enzyme for methionine synthase (MS), and hypotheses for vitamin B12-mediated promotion of CSCs. Abbreviations: SAM (s-adenosylmethionine), THF (tetrahydrofolate), HCY (homocysteine), OSKM (OCT4, SOX2, KLF4, MYC) F. Western blot analysis of CD320, H3K4me3, and H3K36me3 in CD320-overexpressing or control 231-LM2 cells G. CSC frequency in 231-LM2 cells transduced with a CD320-overexpressing (CD320 OE) or control (Ctrl) lentivirus, as measured via Incucyte analysis 36 hours after seeding. Unpaired t test, ns = non-significant. H. Mass spectrometry analysis of methionine and SAM levels in 231-LM2 cells transduced with a CD320-overexpressing or control lentivirus. 2way ANOVA with Tukey’s multiple comparisons test, *p<0.05, **p<0.01 I. Incucyte analysis of 231-LM2 cells cultured in media with or without supplementation with vitamin B12 that was preincubated with human transcobalamin II (B12-TCII) J. Western blot analysis of CD320, H3K4me3, and H3K36me3 in 231-LM2 cells transfected with siCD320 or siControl (siCtrl) RNA. K. Incucyte analysis of CD320 knockdown in 231-LM2 cells. L. Mass spectrometry analysis of methionine and SAM levels in 231-LM2 cells transfected with siCD320 or siCtrl RNA. 2way ANOVA with Tukey’s multiple comparisons test, ns = non-significant. M. Incucyte analysis of 231-LM2 cells treated with 50nM AG-270 50nM or vehicle control N. Mass spectrometry analysis of FACS-isolated 231-LM2 CSCs or nonCSCs. 2way ANOVA with Tukey’s multiple comparisons test, ns = non-significant.

## Description

Cancer stem cells (CSCs) represent a small population of tumor cells that are primarily defined by their ability to initiate tumorigenesis (Al-Hajj et al., 2003). This population of cells has high self-renewal potential and gives rise to the bulk of tumor cells, which have a more differentiated phenotype (nonCSCs) (Loh & Ma, 2024). Importantly, CSCs are highly resistant to chemotherapy and are ultimately responsible for treatment relapse and metastasis (Lytle, Barber, & Reya, 2018). As such, understanding the unique biology of CSCs is crucial for developing more effective therapeutics.

Recent epidemiologic studies have shown a correlation between elevated vitamin B12 levels and increased risk of solid cancer metastasis (Urbanski et al., 2020), and several lines of evidence suggest a potential link between vitamin B12 and the stem phenotype. Vitamin B12 is a coenzyme that is required for methionine synthase activity, which contributes to methionine and S-adenosylmethionine (SAM) generation (Froese, Fowler, & Baumgartner, 2019). Elevation of both SAM and methionine levels have been associated with maintenance of embryonic and pluripotent stem cells, respectively (Shiraki et al., 2014) (Ozawa et al., 2023). Furthermore, vitamin B12 was recently identified as a limiting factor for inducing cellular plasticity in cellular reprogramming studies (Kovatcheva et al., 2023). Vitamin B12 is transported into the cell via the transcobalamin receptor, CD320, which is upregulated early during the induction of pluripotent stem cells (Kovatcheva et al., 2023). We therefore hypothesized that CD320 might be important in induction or maintenance of the CSC phenotype. We utilized an extensively validated fluorescent CSC reporter, which allows us to readily quantitate and isolate CSCs and nonCSCs, to investigate the expression and potential functional role of CD320 in breast CSCs (Sharma et al., 2021; Tang et al., 2015).

We found that expression of CD320 protein is increased (~2.3-fold) in sorted cultures of CSCs compared to nonCSCs in the human triple-negative breast cancer (TNBC) cell line MDA-MB-231-LM2 (‘231-LM2’) ( Figure 1A ). CD320 expression is also increased, to a lesser extent (~1.9-fold), in the human BT549 TNBC cell line ( Figure 1A ). These findings were corroborated by western blot quantification. In addition, flow cytometry confirmed elevated CD320 expression on the cell surface of both cell lines ( Figure 1B, Figure 1C ). To confirm relevance *in vivo,* we assessed surface CD320 expression on CSCs and nonCSCs dissociated from orthotopically implanted 231-LM2 tumors ( Figure 1D ). We observed an even more pronounced difference in CD320 expression in recovered CSCs vs nonCSCs, as well as a new minor subpopulation of CSCs that exhibited extremely elevated CD320 expression *in vivo* . In both cell compartments (CSC and nonCSC), the percentage of CD320+ 231-LM2 cells was higher *in vitro* ( Figure 1B ) than *in vivo* ( Figure 1D ). As CD320 expression is increased during cell division (Gick et al., 2020), this difference may be attributable to the increased proliferation rate of nutrient-replete 2D cultures versus those in 3D models or nutrient-poor tumors (Guerrero-Lopez et al., 2025). Collectively, these findings prompted us to examine the role of CD320 further.

Via methionine generation, vitamin B12 contributes to SAM synthesis ( Figure 1E )(Froese, Fowler, & Baumgartner, 2019). SAM is known for its role as a universal methyl donor in many biological reactions (Lee et al., 2023), including in histone methylation (Du et al., 2015). The stem phenotype is maintained epigenetically, with some histone H3 modifications playing especially important roles. The H3K4me3 methylation mark, which is particularly dependent on methionine, is associated with transcription of the master regulators of stemness (NANOG, POU5F1, and ID1)(Ozawa et al., 2023). The H3K36me3 mark, which is involved in transcriptional fidelity and cellular plasticity (McCauley et al., 2021) (Pashos et al., 2025; Yu et al., 2022; Zhang et al., 2018), was recently shown to be particularly sensitive to vitamin B12 levels (Kovatcheva et al., 2023). In a direct link, vitamin B12 utilization is associated with OCT4, SOX2, KLF4, and MYC (OSKM)-mediated cellular reprogramming to the stem cell state via the H3K36me3 epigenetic mark (Kovatcheva et al., 2023). We hypothesized that SAM generation is increased in CSCs due to increased vitamin B12 uptake via elevated CD320, and that SAM then contributes to CSC induction or maintenance via trimethylation of the H3K4 and H3K36 residues. Thus, while H3K4me3 and H3K36me3 are both known to have diverse biological roles (Wang et al., 2025; Xiao et al., 2021), both potentially contribute to regulating the stem phenotype, so we sought to investigate the impact of CD320 expression on these epigenetic marks and CSC expansion kinetics.

First we used lentivirus transduction to overexpress CD320 in 231-LM2 cells ( Figure 1F- H). CD320 overexpression was confirmed via western blotting ( Figure 1F ). CD320 overexpression did not modulate H3K4me3 or H3K36me3 trimethylation marks ( Figure 1F ). Furthermore, CD320 overexpression did not promote CSC expansion as hypothesized, as evidenced by a similar frequency of CSCs in 231-LM2 cells with and without CD320 overexpression ( Figure 1G ). Using mass spectrometry (MS), we confirmed that CD320 overexpression significantly increased methionine levels as expected, but we saw no effect on SAM levels ( Figure 1H ). Vitamin B12 levels in these samples were below the limit of detection. We also examined the impact of vitamin B12 supplementation on mixed cultures of CSCs and nonCSCs. Addition of vitamin B12 complexed with transcobalamin increased the expansion of both populations, but did not exhibit any preferential impact on CSCs as assessed by Incucyte Live Cell Imaging ( Figure 1I ). The data suggest that vitamin B12, methionine and SAM levels are not limiting for histone methylation at endogenous levels of CD320 expression.

Next, we knocked down endogenous CD320 via transfection with small interfering RNA (siRNA) and confirmed CD320 knockdown via western blotting ( Figure 1J ). CD320 knockdown had little effect on either H3K4me3 or H3K36me3 epigenetic marks ( Figure 1J ). Furthermore, CD320 knockdown had minimal effect on CSC expansion *in vitro* , as measured via the frequency of CSCs quantified via Incucyte live cell imaging for 231-LM2 cells ( Figure 1K ). TAZ knockdown, which has previously been shown to reduce CSC frequency (Tang et al., 2025), was included as a positive control. Reducing CD320 expression had no effect on methionine or SAM levels ( Figure 1L ) suggesting that CD320 may not be the rate-limiting component for generation of these metabolites in these breast cancer models.

The data suggest that the level of CD320 expression does not significantly modulate intracellular SAM levels in the 231-LM2 cell line, which likely explains the lack of effect on H3K4me3 or H3K36me3. MAT2A is the rate limiting enzyme involved in the generation of SAM from methionine and ATP. To further investigate if SAM affects 231-LM2 CSC expansion independently of CD320 expression, we treated cells with the MAT2A inhibitor AG-270 (Gounder et al., 2025; Konteatis et al., 2021). There was no effect of AG-270 on CSC representation ( Figure 1M ), suggesting SAM levels do not affect 231-LM2 CSC expansion *in vitro* regardless of CD320 expression. This conclusion was corroborated by the observation that 231-LM2 CSCs and nonCSCs exhibit similar levels of SAM and methionine despite differing CD320 expression ( Figure 1N ).

Despite the known involvement of vitamin B12 in SAM generation, and a proven role for SAM in promoting stemness in other systems, this data collectively suggests that neither CD320 expression nor SAM generation has an effect on breast CSC expansion in the two models studied here. It is important to note that, while significant, the differences in expression of CD320 in CSC vs nonCSCs *in vitro* were small compared to the differences we observed *in vivo* ( Figure 1A- B vs Figure 1C ). Investigation into the impact of CD320 expression by CSCs *in vivo* may still be warranted.

## Methods

**Cell Lines and culturing conditions**

MDA-MB-231-LM2 (“231-LM2”) was cultured in low glucose DMEM (Thermo) supplemented with 10% FBS. BT549 cells were cultured in DMEM (Thermo) supplemented with 10% FBS and 0.023U/ml human recombinant insulin (Gibco). All cells were grown at 37°C with 5% CO2 and underwent authentication via short tandem repeat profiling (TransnetYX Inc, CA).

**SORE6 Reporter Infections**

Cell lines were transduced with a constitutively active nuclear GFP marker lentivirus (EF1alpha>nucGFP, Sartorius BA-04888) and selected by FACS. Cells were subsequently infected with the SORE6>dsmCherry pro-lentiviral reporter construct (18065-M02-412) or minCMV>dsmCherry control construct (18065-M04-412) at an MOI of 1 for 48h with 7.5ug/ml polybrene (Sigma) (Tang 2025). CD19+ cells (Biolegend 392504) were isolated via FACS. The dsmCherry is FLAG-tagged for detection by Western blot.

**Western blotting**

CSCs (mCherry+) and nonCSCs (mCherry-) cells were FACS sorted from SORE6-infected cell lines, cultured for 24-48hrs, and lysates harvested via scraping into RIPA (Thermo) containing protease and phosphate inhibitors (Thermo). Equal amounts of protein were subjected to SDS-PAGE on a 12% Mini-PROTEAN TGX gel (Biorad) and electrotransferred to PVDF membranes (Biorad). Membranes were blocked in 5% Blotting-Grade Blocker (Biorad) and probed with antibodies against CD320 (Thermo 10343-1-AP), FLAG (Biolegend 637301), H3K4me3 (CST9751), H3K36me3 (CST4909S) and β-actin (CST3700S). Secondary antibodies (CST 7074S, CST 7076S or Thermo 31470) were added to the membranes and immunoreactive proteins were detected using the SuperSignal West Femto Chemiluminescence kit (Thermo) according to the manufacturer’s instructions. Membranes were washed 3X in between staining, and stripped (Reblot Plus, Sigma) and washed between primary antibody probing.

**Flow cytometric analysis of cell cultures**

Equal numbers of plated cells were detached with trypsin, washed, resuspended in Fc block (Biolegend 422302), and incubated with 0.1ug CD320 antibody (Thermo 10343-1-AP) for 30 minutes at RT. Cells were washed 2X and stained with secondary antibody (Thermo A-31573) for 30 minutes at room temperature (RT). Cells were washed 2X and data acquired on a BD SORPI flow cytometer and analyzed using FlowJo Analysis software.

**Animal model, tumor dissociation, and flow cytometric analysis**

All animal experiments were conducted in compliance with institutional guidelines and under protocol LC-070, approved by the Institutional Animal Care and Use Committee (IACUC) of the Center for Cancer Research (CCR), National Cancer Institute. Virgin female NCI Ath/Nu++ immunodeficient nude mice were obtained from NCI Charles River Labs at 4-6 weeks of age. 500,000 231-LM2 cells in 1:1 DMEM media and Matrigel (Corning) were injected into left and right 4th MFPs. After 6 weeks, tumors were dissociated using the GentleMACS mouse tumor dissociation kit (Milteny Biotec) per the manufacturers instructions, and CD19+ tumor cells isolated via magnetic separation (Milteny Biotec). Cells were stained with Zombie Aqua (Biolegend), followed by staining for CD320 (Thermo 10343-1-AP) using an AlexaFluor647-labeled secondary antibody (Thermo A-31573) for detection. Cells were fixed and permeabilized (BD) and stained for mCherry (Thermo M11217) using an AlexaFluor568-labeled secondary antibody (Thermo A-11077). Data was acquired and analyzed as above.

**Incucyte Live Cell Imaging**

An Incucyte S3 Live-Cell Imaging system with 440/480 ex/em (green) and 565/620 ex/em (red) filters was used to monitor CSC and nonCSC dynamics in culture over time. 50,000 cells were seeded in 24-well tissue culture plates and images captured in the red and green channels every 3h for the duration of the experiment. Images were analyzed using the basic analyzer (Incucyte 2023 Rev B software). Cells expressing mChery above a pre-defined threshold were classified as CSCs, using the minCMV>dsmCherry control (<0.5% of total cells were mCherry+ in the control sample). Cells expressing only GFP were considered nonCSCs.

**CD320 Overexpression**

300,000 231-LM2 were transduced in 12-well plates by exposure to CD320 lentivirus (Origene RC200073L3V) or empty control (Origene PS100092V) at an MOI of 1 or 2 for 48h with 7.5ug/ml polybrene (Sigma). Control or cells overexpressing CD320 were FACS isolated and plated for Incucyte imaging, western blot, or MS analysis.

**Mass spectrometry (MS) sample preparation and analysis**

300,000 cells were seeded a day prior to harvesting, after which they were washed, scraped into ice-cold PBS, centrifuged at 15,000 x g and snap frozen in liquid nitrogen. Prior to MS analysis, cell pellets were lysed by sonication in water and acetonitrile extraction performed. MS analysis was performed on a SCIEX X500B quadrupole time-of-flight mass spectrometer (Framingham, MA) in-line with a Luna Omega 1.6 μM C18 100Å column (Phenomenex, Torrance, CA). Quantitation was performed by multiple reaction monitoring using 150-61 and 399-250 transitions for L-methionine and S-adenosyl methionine, respectively.

**CD320 Knockdown**

250,000 cells were seeded, and the following day, transfected with 10nM siRNA (Horizon Discovery) in OPTI-MEM (Gibco) and RNAiMax (Thermo) for 72hrs. Cells were harvested for Incucyte live cell imaging, western blot, or MS analysis.

**MAT2A inhibition**

1 day after seeding in normal media, 231-LM2 cells were treated with 50nM AG-270 (MedChemExpress) or vehicle control and imaged with the Incucyte.

**Vitamin B12-Transcobalamin II Supplementation**

231-LM2 cells were seeded in normal media with or without supplementation with vitamin B12 (Sigma) preincubated with human transcobalamin II (R&D Systems) for 1hr at RT and imaged with the Incucyte.

**Statistical Analysis**

Statistical tests were performed in GraphPad Prism (version 9.3.1 (471)).
